# Shot noise generated by graphene *p*–*n* junctions in the quantum Hall effect regime

**DOI:** 10.1038/ncomms9068

**Published:** 2015-09-04

**Authors:** N. Kumada, F. D. Parmentier, H. Hibino, D. C. Glattli, P. Roulleau

**Affiliations:** 1 NTT Basic Research Laboratories, NTT Corporation, 3-1 Morinosato-Wakamiya, Atsugi 243-0198, Japan; 2 Nanoelectronics Group, Service de Physique de l'Etat Condensé, IRAMIS/DSM (CNRS URA 2464), CEA Saclay, F-91191 Gif-sur-Yvette, France

## Abstract

Graphene offers a unique system to investigate transport of Dirac Fermions at *p*–*n* junctions. In a magnetic field, combination of quantum Hall physics and the characteristic transport across *p*–*n* junctions leads to a fractionally quantized conductance associated with the mixing of electron-like and hole-like modes and their subsequent partitioning. The mixing and partitioning suggest that a *p*–*n* junction could be used as an electronic beam splitter. Here we report the shot noise study of the mode-mixing process and demonstrate the crucial role of the *p*–*n* junction length. For short *p*–*n* junctions, the amplitude of the noise is consistent with an electronic beam-splitter behaviour, whereas, for longer *p*–*n* junctions, it is reduced by the energy relaxation. Remarkably, the relaxation length is much larger than typical size of mesoscopic devices, encouraging using graphene for electron quantum optics and quantum information processing.

## Abstract (web summary)

Dirac fermions at a *p*–*n* junction can exhibit a wide variety of unusual properties. Here, the authors investigate the dynamics of such fermions in a graphene junction using shot noise measurements and demonstrate the crucial role of junction length.

## Graphical abstract

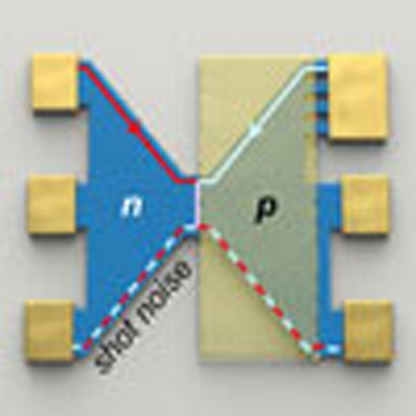

In graphene, owing to the linear and gapless band structure, *n*-type (electron-like) and *p*-type (hole-like) regions can adjoin without a gap in between. Investigations of charge carrier transport across such a *p*–*n* junction (PNJ) have revealed unique phenomena reflecting the Dirac Fermion character in graphene, such as Klein tunneling^1^,^2^,^3^, Veselago lensing^4^ and snake state^5^,^6^. In the quantum Hall (QH) effect regime under high magnetic field *B*, the conductance across a PNJ shows plateaus at *G*_PNJ_=*G*_0_|*ν*_1_||*ν*_2_|/(|*ν*_1_|+|*ν*_2_|), where *G*_0_=*e*^2^/*h* is the conductance quantum (*h* is Planck's constant), *ν*_1_=2, 6, 10, … and *ν*_2_=−2, −6, −10, … are the Landau level filling factor in the *n* and *p* regions, respectively^7^,^8^,^9^. This conductance quantization in bipolar QH states has been explained by the mixing of counter-circulating electron and hole edge modes^10^,^11^: the current injected to the PNJ is distributed to electron and hole modes in the PNJ by the mode mixing with the ratio depending on the number of each modes, thus on *ν*_1_ and *ν*_2_, and then partitioned at the exit of the PNJ (Fig. 1b). This process gives rise to the conductance quantization at the values depending on *ν*_1_ and *ν*_2_. However, experimental study of the mode-mixing mechanism is lacking. If the mode mixing is caused by quasielastic scattering as suggested in ref. 10, 11, a graphene PNJ acts as a beam splitter of electrons and holes. A better understanding of these properties is a crucial step towards the development of electron quantum optics experiments in graphene; beam splitters together with edge states are key components for electronic interferometry^12^,^13^,^14^.

Shot noise measurements can provide insight into the mode-mixing mechanism (Supplementary Figs 1 and 4): when the electron and hole modes biased by *V*_sd_ are mixed, the energy distribution in the PNJ *f*_PNJ_(*E*) becomes out-of-equilibrium and the subsequent partitioning of the modes gives rise to the shot noise. If the mode mixing is quasielastic, *f*_PNJ_(*E*) is a double-step function. At zero temperature, the shot noise generated by the partitioning of the modes with double-step *f*_PNJ_(*E*) is expected to be (10, Supplementary Note 1),


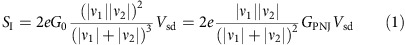


characterized by the Fano factor *F*=*S*_I_/2*eI*, yielding *F*=0.25 for (ν_1_, ν_2_)=(2, −2). Energy losses towards external degrees of freedom can drive *f*_PNJ_(*E*) towards a Fermi distribution with a chemical potential *eV*_sd_/2 (Fig. 1c)^15^,^16^,^17^, causing the noise (and thus the Fano factor) to vanish as the carrier dwell time in the PNJ becomes larger than the energy relaxation time (18). Inelastic processes between modes in the PNJ may occur, causing *f*_PNJ_(*E*) to relax towards a Fermi distribution with a finite temperature *T*_eff_(*V*_sd_) given by the balance between the Joule power dissipated in the PNJ and the heat flowing along the outgoing electronic channels^10^,^19^ (Supplementary Note 1). In this case, the Fano factor becomes 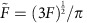 [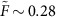 for (*ν*_1_, *ν*_2_)=(2, −2)]. Note that standard transport measurements yield the same value of *G*_PNJ_ for all cases, and thus cannot distinguish them.

In the following, we investigate the evolution of *f*_PNJ_(*E*) with carrier dwell time in the PNJ, which can be tuned by changing the length of the PNJ. We demonstrate that the amplitude of the noise is consistent with an electronic beam-splitter behaviour when PNJ is short and the energy losses are negligible.

## Results

### Measurement set-up

**Figure 1:**
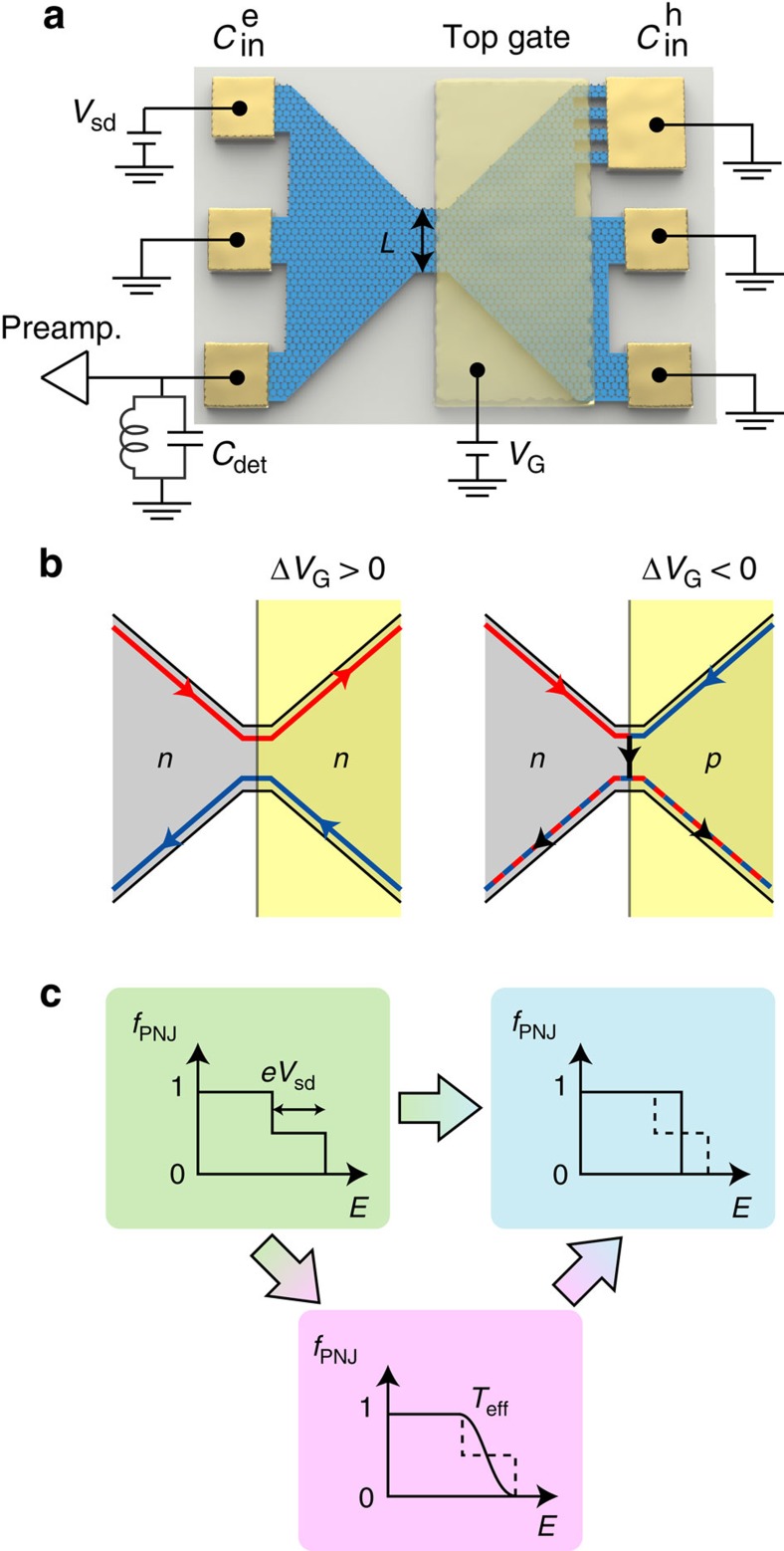
Schematic of the device. (**a**) The top gate covers the right half of the graphene. In the ungated region, the carrier type is electron and the density is fixed at about 5 × 10^11^ cm^−2^. In the gated region, the carrier type can be changed to hole by applying negative gate voltage *V*_G_. A source–drain bias *V*_sd_ is applied to either 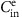 or 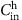 and the noise is measured on *C*_det_. The upperright contact is comb shaped to achieve a good contact to the *p* region (Supplementary Methods). (**b**), QH edge modes for the unipolar (left) and bipolar regimes (right). (**c**), *f*_PNJ_(*E*) in the quasielastic case (left). In the presence of energy relaxation towards external degrees of freedom, *f*_PNJ_(*E*) becomes a Fermi distribution at base temperature (right). Additional inelastic scattering between modes in the PNJ may drive towards a Fermi distribution at finite temperature *T*_eff_ (bottom), which can then relax towards a cold Fermi distribution.

We obtained bipolar graphene devices using a top gate covering half of the graphene (Fig. 1a); the carrier type in the gated region can be tuned by the gate voltage *V*_G_, while that in the ungated region is fixed to electron by the doping (Methods). Therefore, the PNJ is formed at the interface between the gated and ungated regions when the carrier type in the gated region is hole for Δ*V*_G_≡*V*_G_−*V*_CNP_<0 (*V*_CNP_ is the gate voltage at the charge neutrality point). We prepared five samples with different interface lengths *L*=5, 10, 20, 50, and 100 μm. The direction of *B* is chosen so that electron and hole modes from the ohmic contacts 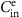 and 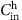, respectively, merge at the PNJ. For the noise measurement, *V*_sd_ is applied to either 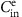 or 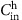 and the noise is detected on *C*_det_ (Fig. 1a; Supplementary Methods and Supplementary Fig. 5). Magnetic fields up to *B*=16 T have been applied. The base temperature is *T*=4.2 K.

### Detection of shot noise generated at PNJ

**Figure 2:**
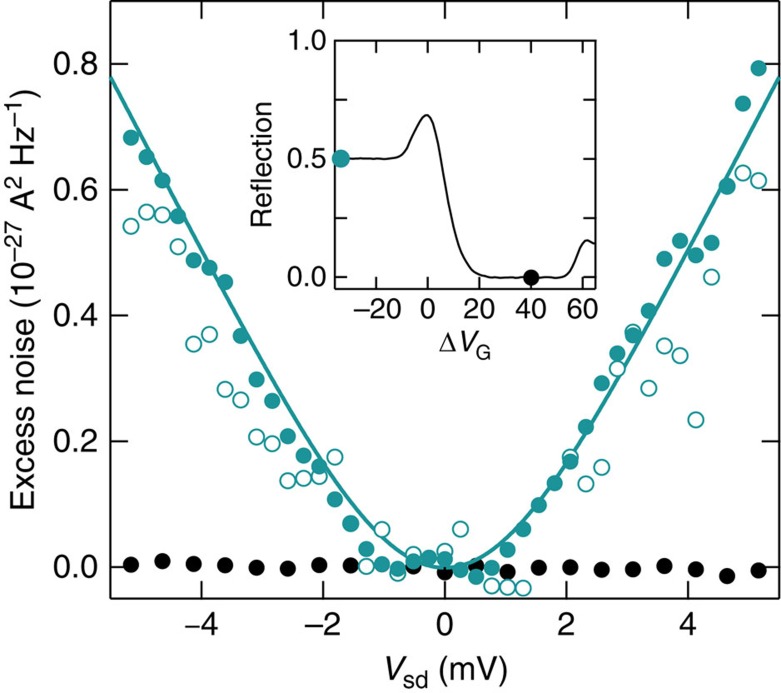
Shot noise generated by *p*–*n* junction. Excess noise Δ*S*_I_ of the sample with *L*=50 μm as a function of *V*_sd_ for the bipolar QH state at (*ν*_ug_, *ν*_g_)=(2, −2) (cyan circles) and the unipolar QH state at (*ν*_ug_, *ν*_g_)=(2, 2) (black circles). For the solid and open circles, *V*_sd_ is applied to 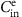 and 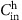, respectively. The solid trace is the result of a fit using equation (2). Inset: reflection of the average current from 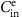 to *C*_det_ as a function of Δ*V*_G_. The cyan and black circles represent the Δ*V*_G_ at which the noise is measured.

The inset of Fig. 2 shows the reflection of the averaged current from 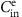 to *C*_det_ in the sample with *L*=50 μm. The magnetic field is *B*=10 T, at which the filling factor in the ungated region is fixed at *ν*_ug_=2. When the bipolar QH state at (*ν*_ug_, *ν*_g_)=(2, −2) is formed for Δ*V*_G_<−10 V, the current injected from 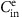 is partitioned equally to the electron and hole modes at the exit of the PNJ, yielding a reflection of 1/2. A current noise *S*_I_ appears in this regime. As *V*_sd_ applied to 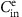 is increased, the excess noise Δ*S*_I_≡*S*_I_−*S*_I_(*V*_sd_=0) increases (solid cyan circles in Fig. 2). Δ*S*_I_ approaches linear behaviour for *eV*_sd_>*k*_B_*T*, characteristic of the shot noise. A similar signal appears when *V*_sd_ is applied to 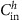 (open cyan circles). In the unipolar QH state at (*ν*_ug_, *ν*_g_)=(2, 2) for 20<Δ*V*_G_<50 V, on the other hand, the shot noise is zero (solid black circles), proving that the shot noise is indeed generated at the PNJ.

Quantitatively, we extracted *F* by fitting Δ*S*_I_ as a function of *V*_sd_ using the relation including temperature broadening^20^:


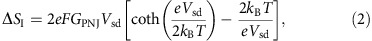


where *G*_PNJ_ is obtained by average current measurements. The fit yields *F*=0.015, which is one order of magnitude smaller than *F*=0.25 expected for the noise from the double-step energy distribution. This indicates that *f*_PNJ_(*E*) evolves during the charge propagation for *L*=50 μm, reducing the shot noise.

### Evolution of noise amplitude with PNJ length

**Figure 3:**
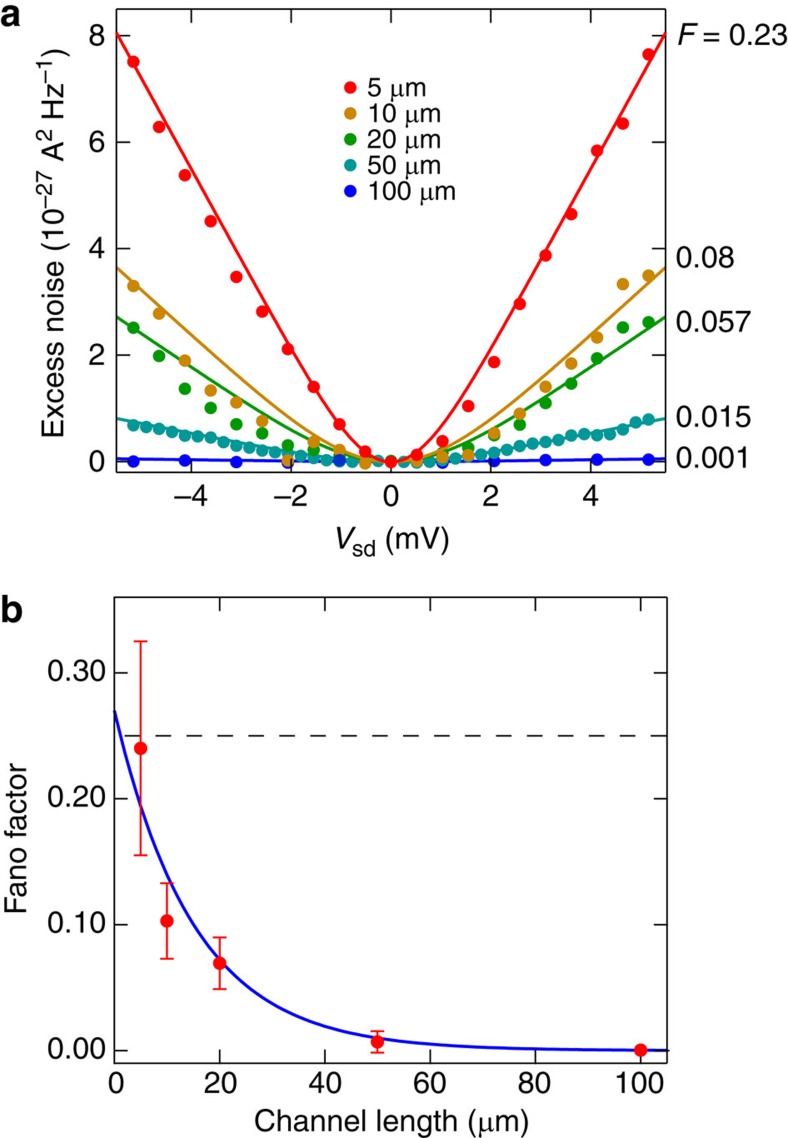
Shot noise as a function of *p*–*n* junction length. (**a**) Δ*S*_I_ in the bipolar QH state at (*ν*_ug_, *ν*_g_)=(2, −2) as a function of *V*_sd_ for the five samples with *L* between 5 and 100 μm. All data are taken at *B*=10 T. Lines are results of the fit using equation (2), by which the values of *F* (indicated on the right-hand side of the figure) are obtained. (**b**), *F* as a function of *L*. The error bars represent the s.d. of the extracted *F* for different values of *B* and Δ*V*_G_ in the bipolar QH state (an example is shown in Fig. 4c). An exponential fit (blue curve) yields a relaxation length *L*_0_=15 μm. The dashed horizontal line represents the expected value *F*=0.25 for the double-step *f*_PNJ_(*E*).

The evolution of *f*_PNJ_(*E*) can be investigated using samples with different *L*. Figure 3a shows the results of the noise measurement in the bipolar QH state at (*ν*_ug_, *ν*_g_)=(2, −2) for the five samples with *L* between 5 and 100 μm. The data show that the shot noise decreases with increasing *L* and almost disappears at *L*=100 μm (Fig. 3b), indicating that *f*_PNJ_(*E*) relaxes to the thermal equilibrium through interactions with external degrees of freedom. An exponential fit of the data yields a relaxation length *L*_0_=15 μm. The extrapolation to *L*=0 gives *F*∼0.27, consistent with the limit of quasielastic scattering *F*=0.25. Furthermore, the decrease is well reproduced by a model gradually coupling the modes propagating in the PNJ to cold external states (Supplementary Note 1 and Supplementary Fig. 2). Note that we are not able to observe whether inelastic scattering occurs inside the PNJ, because of the large error bars explained below. An important implication of the results is that, within the typical scale of usual mesoscopic devices (<1 μm), the energy loss towards external degrees of freedom is negligible and the current channels in the PNJ can be regarded as an isolated system.

### Fluctuation of noise

**Figure 4:**
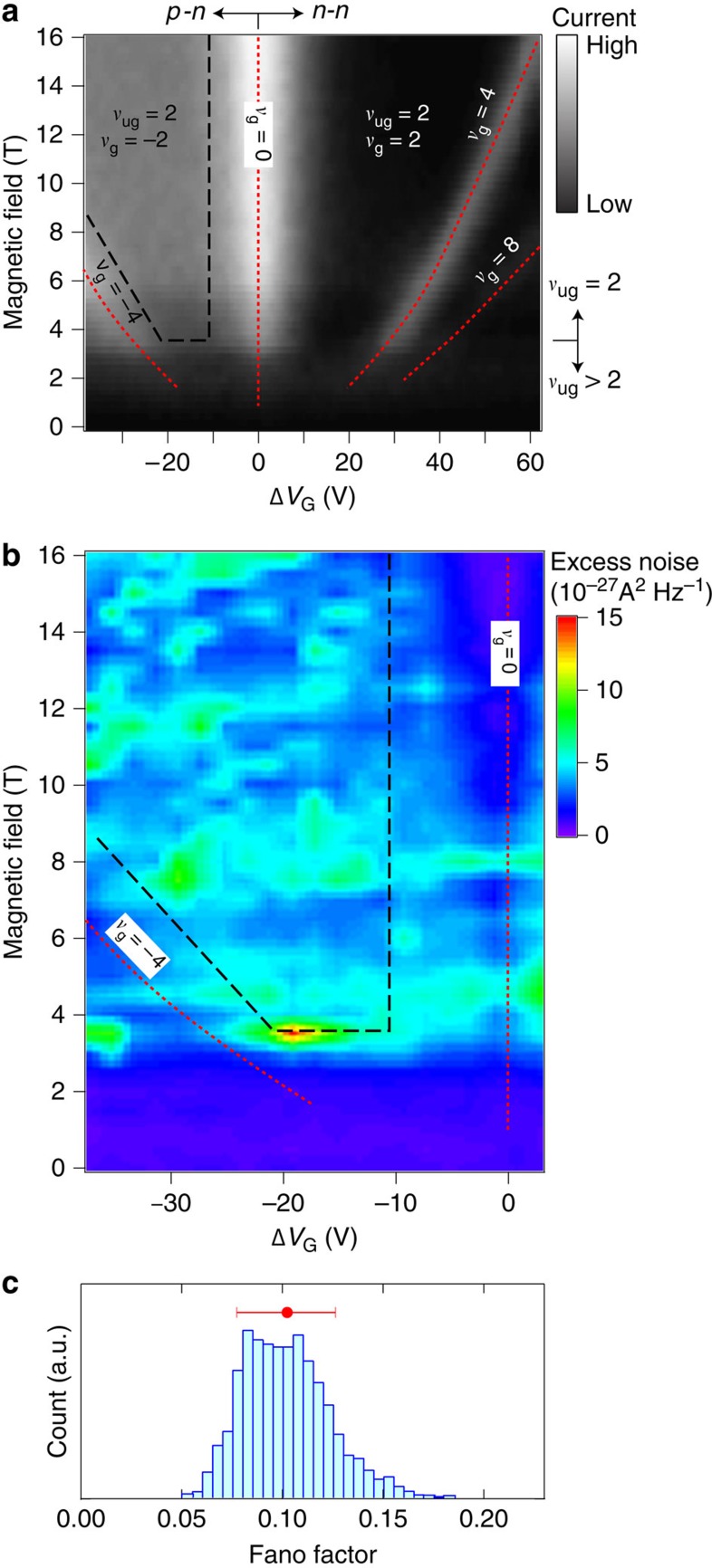
Fluctuations of shot noise. (**a**) Grey scale plot of the current from 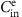 to *C*_det_ at 2 kHz for the sample with *L*=10 μm. The current is measured through the resonator and the amplifier. Black dashed lines indicate the region for the bipolar QH state at (*ν*_ug_, *ν*_g_)=(2, −2). Red dotted lines represent the non-QH state in the gated region at *ν*_g_=−4, 0, 4 and 8. (**b**) Colour scale plot of Δ*S*_I_ for the sample with *L*=10 μm as a function of Δ*V*_G_ and *B*. The applied current is fixed at 400 nA, which corresponds to *V*_sd_=5.2 mV in the bipolar QH state at (*ν*_ug_, *ν*_g_)=(2, −2). The black dashed and red dotted lines correspond to those in **a**. (**c**) Histogram of the Fano factor within the bipolar QH state. The red dot and bar represent the mean value and the s.d., which correspond to the data point and the error bar in Fig. 3b, respectively.

We further investigate the properties of the PNJ focusing on the energy relaxation mechanism by measuring the shot noise for a wide range of *B* and Δ*V*_G_. We identify the electronic states in the gated and ungated regions as a function of *B* and Δ*V*_G_ by a low-frequency current measurement from 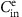 to *C*_det_ (Fig. 4a) and then investigate the relation between those states and the noise. The electronic state in the ungated region depends only on *B* and the *ν*_ug_=2 QH state is formed for *B*>4 T. In the gated region, the non-QH states at *ν*_g_=8, 4, 0 and −4 appear as a current peaks. The bipolar QH state at (*ν*_ug_, *ν*_g_)=(2, −2) is formed for *B*>4 T and between *ν*_g_=0 and −4 (the region indicated by dashed lines), in which the current is almost constant, consistent with the quantized conductance^7^,^8^,^9^. The shot noise in the sample with *L*=10 μm becomes small (Fig. 4b) when either or both ungated and gated regions are in a non-QH state. This confirms that shot noise is generated by the PNJ in a well-developed bipolar QH state. Within the bipolar QH state, the shot noise fluctuates largely, depending on *B* and Δ*V*_G_. This noise fluctuation cannot be ascribed to *G*_PNJ_, which is almost constant in the bipolar QH state. Furthermore, since the noise is generated in the well-developed QH state, the existence of multiple noise sources is unlikely. These facts indicate that the noise fluctuation is due to the fluctuation of the energy relaxation rate, which induces the fluctuation of Fano factor. Figure 4c shows the histogram of the Fano factor in the bipolar QH state calculated using equation (2). The s.d. is about 50% of the mean value.

## Discussion

The random variation of the energy relaxation rate as a function of *B* and Δ*V*_G_ suggest that localized states in bulk graphene play a main role for the energy relaxation. Energy in the PNJ can escape to the bulk graphene through Coulomb interaction with localized states: high frequency potential fluctuations in the PNJ, which is the source of the shot noise, are dissipated in the localized states. Since the energy level and the profile of the localized states depend on *B* and Δ*V*_G_, fluctuations of the relaxation rate can be induced. On the other hand, the average current through the PNJ, which merely reflects the transmission coefficient, is hardly affected by the localized states. Note that a simple model of interaction with two-dimensional phonons in the PNJ fails to quantitatively reproduce our observations (Supplementary Note 1 and Supplementary Fig. 3). It is reported that the electron–phonon coupling is expected to be vanishingly small in usual unipolar edge channels^21^,^22^,^23^. To understand the energy relaxation length quantitatively, detailed analysis including interactions with phonons and any other possible mechanisms for the energy relaxation is necessary.

In conclusion, we showed that the mode mixing at PNJ in graphene bipolar QH states leads to non-equilibrium *f*_PNJ_(*E*), generating shot noise. For a short PNJ (*L*<<15 μm), the energy loss towards external states is negligible and the noise is consistent with a quasielastic mode mixing. This suggests that a graphene PNJ can act as a beam splitter. Since 15 μm is much larger than typical length scale of mesoscopic devices, our results encourage using graphene for electron quantum optics experiments and quantum information.

## Methods

### Device fabrication

We prepared a graphene wafer by thermal decomposition of a 6H-SiC(0001) substrate. SiC substrates were annealed at around 1,800 °C in Ar at a pressure of <100 torr. For the fabrication of devices, graphene was etched in an O_2_ atmosphere. After the etching, the surface was covered with 100-nm-thick hydrogen silsesquioxane (HSQ) and 60-nm-thick SiO_2_ insulating layers. As a result of doping from the SiC substrate and the HSQ layer, graphene has *n*-type carriers with the density of about 5 × 10^11^ cm^−2^. The width of the PNJ roughly corresponds to the thickness of the insulating layers and estimated to be 200 nm at most. In the QH effect regime, because of the Landau level quantization, the width becomes smaller with *B*. An important advantage of the SiC graphene is its size: it is single domain for 1 cm^2^, allowing us to investigate the effect of PNJ length.

### Noise measurement

For the noise measurement, the current noise is converted into voltage fluctuations across one 2.5 kΩ resistor in series with the sample. A 500-kHz bandwidth 3-MHz tank circuit combined with a homemade cryogenic amplifier is used. After further amplification and digitization, the autocorrelation voltage noise spectra is calculated in real-time by a computer. Accurate calibration of the noise is done using Johnson–Nyquist noise that relies on the quantification of the resistance at *ν*=2 and the temperature of the system.

## Additional information

**How to cite this article**: Kumada, N. *et al*. Shot noise generated by graphene *p*–*n* junctions in the quantum Hall effect regime. *Nat. Commun*. 6:8068 doi: 10.1038/ncomms9068 (2015).

## Supplementary Material

Supplementary Information Supplementary Figures 1-5, Supplementary Note 1, Supplementary Methods and Supplementary References
