# Supplementary material for: Shot noise generated by graphene p–n junctions in the quantum Hall effect regime
Source: Nat Commun. 2015 Sep 4;6:8068. doi: 10.1038/ncomms9068 (PMC5426518; doi:10.1038/ncomms9068)
Supplement: Supplementary Information — Supplementary Figures 1-5, Supplementary Note 1, Supplementary Methods and Supplementary References [file ncomms9068-s1.pdf]

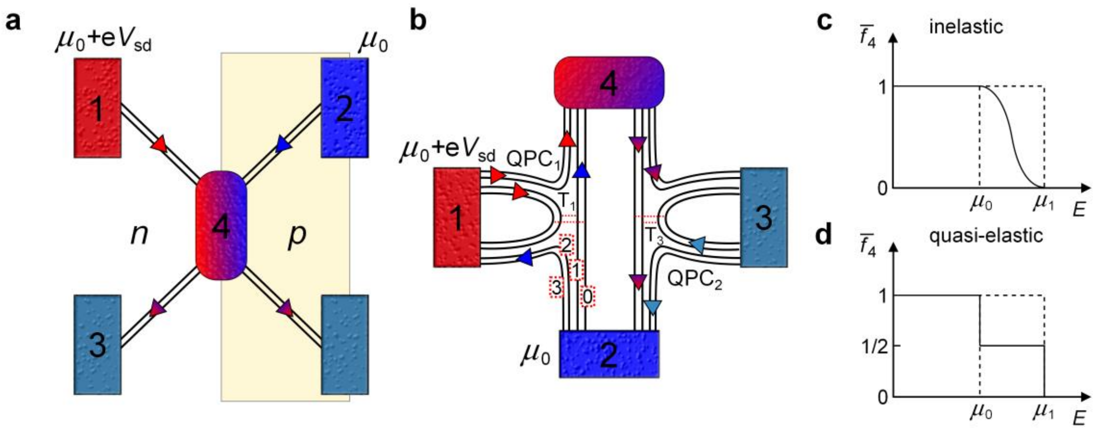

Supplementary Figure 1 | Mode mixing by inelastic and quasi-elastic scattering. a, Schematic representation of a graphene PNJ in the QH state at $(\nu_{ug}, \nu_g) = (2, -2)$. The edge channels mix in the fictitious contact 4. **b**, Four-terminal configuration used to mimic inelastic and quasi-elastic scattering in a PNJ, adapted from (1). **c** and **d**, Average energy distribution function in the fictitious contact in the inelastic and quasi-elastic cases, respectively.

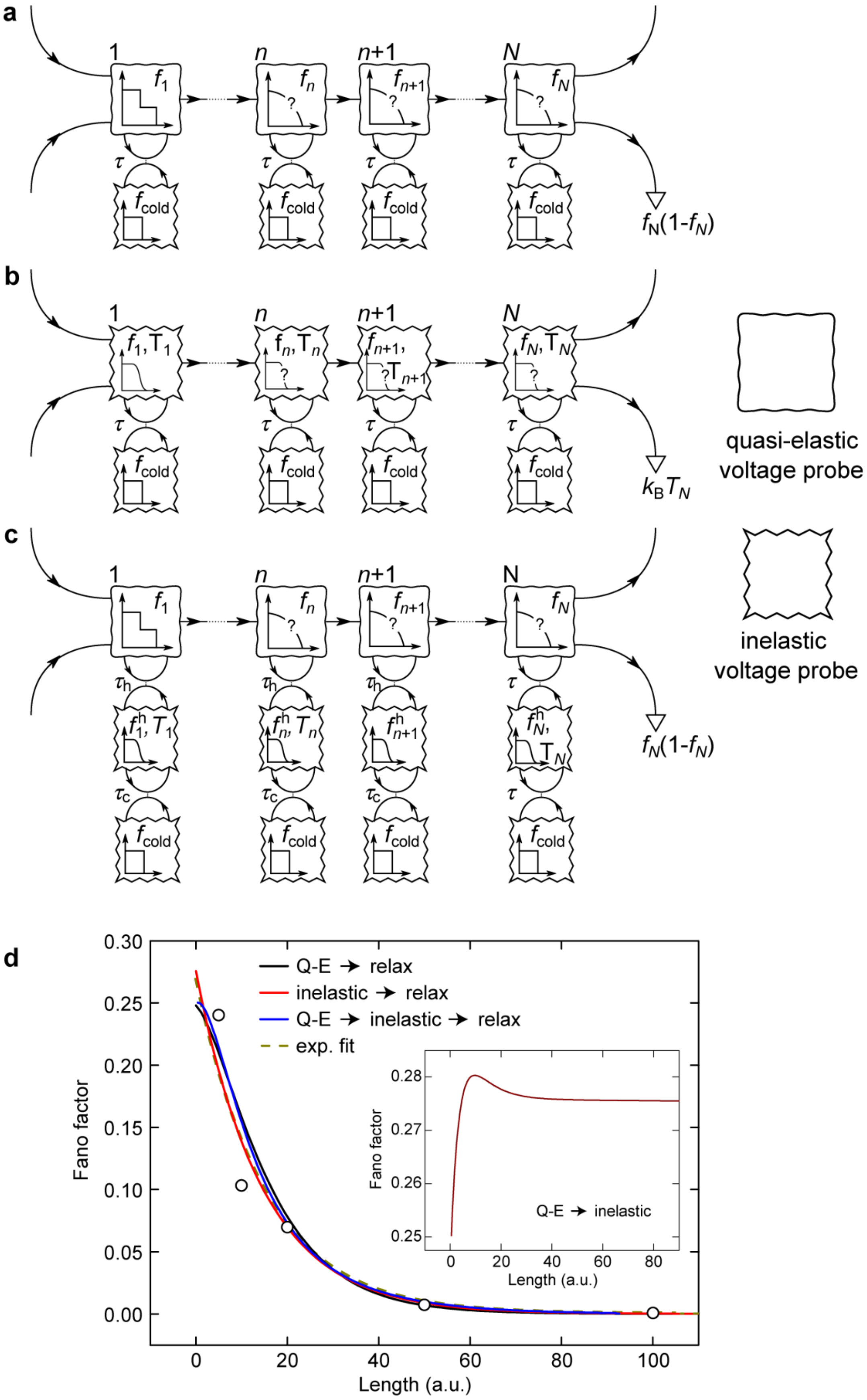
a
1
n
n+1
N
f1
fn
fn+1
fN
τ
fcold
fN(1-fN)
b
f1, T1
fn, Tn
fn+1, Tn+1
fN, TN
kB TN
quasi-elastic voltage probe
c
τh
τc
f1h, T1
fnh, Tn
fn+1h
fNh, TN
inelastic voltage probe
d
Q-E → relax
inelastic → relax
Q-E → inelastic → relax
exp. fit
Fano factor
Length (a.u.)
0.30
0.25
0.20
0.15
0.10
0.05
0.00
0
20
40
60
80
100
Q-E → inelastic
0.28
0.27
0.26
0.25

Supplementary Figure 2 | Models for the energy relaxation a, Quasi-elastic scattering model with direct energy loss towards cold external states. **b**, Fully inelastic scattering model with direct energy loss towards cold external states. **c**, Quasi-elastic scattering model with energy relaxation both between modes propagating in the PNJ and cold external states. In all three panels, soft corrugated squares symbolize quasi-elastic contacts with an out-of-equilibrium energy distribution function determined by energy-conserving current balance, while hard corrugated squares symbolize inelastic voltage probe with equilibrium Fermi distribution functions at temperature either given by power balance or fixed to zero. **d**, Fano factor in all three models plotted as a function of the length in arbitrary units corresponding to the number of fictitious contacts. Open circles correspond to the experimental data shown in the main text. Inset: Fano factor as a function of length for a variant of the third model where energy transfers towards cold external states are suppressed ($\tau_c = 0$). The Fano factor gradually goes from 1/4 to ~0.28.

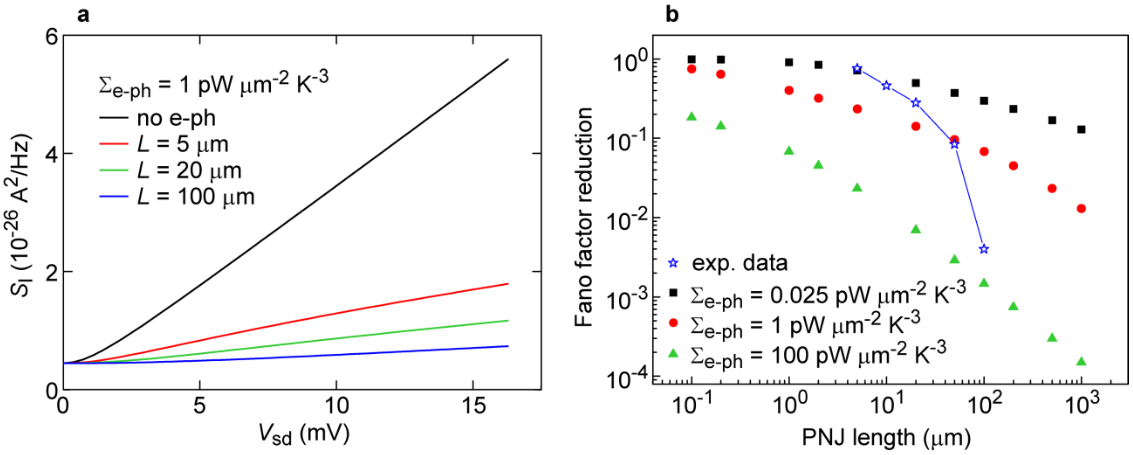

Supplementary Figure 3 | Effect of electron-phonon coupling a, Calculated current noise versus bias voltage, in absence of electron-phonon coupling (black line), and in presence of finite electron-phonon coupling ($\Sigma_{\mathrm{e-ph}} = 1\,\mathrm{pW}\,\mu\mathrm{m}^{-2}\,\mathrm{K}^{-3}$) for $\mathrm{L} = 5, 20, 100\ \mu\mathrm{m}$. **b**, Log-log scale evolution of the slope of the noise (with respect to the Fano factor in absence of electron-phonon coupling) with the PNJ length for several values of $\Sigma_{\mathrm{e-ph}}$. The blue stars represent the experimental results shown in the main paper.

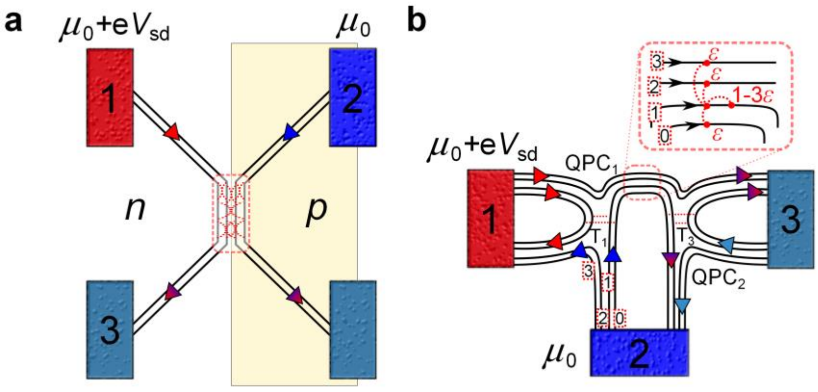

Supplementary Figure 4 | Mode mixing by coherent scattering. a, Schematic representation of a graphene PNJ in the QH state at $(\nu_{\mathrm{ug}}, \nu_{\mathrm{g}}) = (2, -2)$. The edge channels are coupled in the PNJ region through coherent scattering processes. **b**, Three-terminal configuration used to mimic coherent scattering in a PNJ, adapted from (1). The zoom on the central region defines the squared scattering amplitudes between copropagating edge channels.

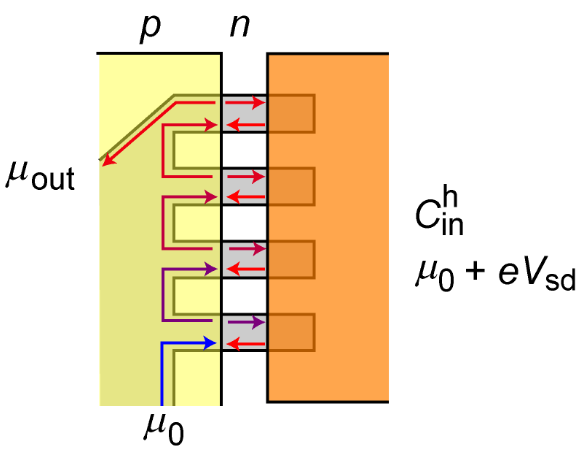

Supplementary Figure 5 | Contact to hole edge channels, Schematic illustration of the device structure around $C_{\mathrm{in}}^{\mathrm{h}}$. Current flow between hole edge channels and $C_{\mathrm{in}}^{\mathrm{h}}$ is indicated by the arrows. The colors of the arrows represent the energy equilibration of the hole edge channels to $C_{\mathrm{in}}^{\mathrm{h}}$.

Supplementary note 1 | Theoretical background

In this section, we present some of the theoretical elements used to analyze our data. These elements are mainly based on (1), and describe the equilibration of edge channels in the PNJ in terms of inelastic and quasi-elastic scattering occurring between the edge channels in the PNJ. We then present various models of energy relaxation to account for the effect of the length of the junction, including energy losses towards phonons. Finally, for the sake of completeness, we mention a model of fully coherent scattering between the edge channels propagating in the PNJ, which does not agree with the experimental data.

Inelastic and quasi-elastic scattering

In this part, we discuss the effects of inelastic and quasi-elastic scattering between copropagating edge states on transport and noise. We consider the mixing of electron and hole edge modes with the chemical potential $\mu_0 + eV_{\mathrm{sd}}$ and μ_0, respectively, at $(\nu_{\mathrm{ug}}, \nu_{\mathrm{g}}) = (2, -2)$. To model inelastic and quasi-elastic scattering, we add a fictitious contact (noted 4 in Supplementary Figure 1**a**) in the central (PNJ) region, where the mode mixing occurs. To calculate the current and the noise, we use the formalism introduced in (1), applied to the four-terminal configuration shown in Supplementary

Figure 1**b**: a first quantum point contact (noted QPC1) is used to obtain four copropagating edge channels towards contact 4 (which mimics the PNJ), where two channels stem from contact 1 (thus mimicking the incoming channels in the *n*-region) and two channels stem from contact 2 (mimicking the incoming channels in the *p*-region). A second QPC (QPC2) is then used to divert two of the channels leaving contact 4 towards the contact 3 (mimicking the outgoing channels in the *n*-region) and the other two towards the contact 2 (mimicking the outgoing channels in the *p*-region). Note that to achieve this configuration, one has to use four edge channels (yielding a total "virtual" filling factor of 4) and set the transmissions of the two innermost channels through both QPCs to zero. Doing so fully satisfies the important condition stated above, and gives a completely equivalent description of the system shown in Supplementary Figure 1**a**.

The noise spectrum is defined as $S_{\alpha,\beta}(\omega)2\pi\delta(\omega+\omega') = \langle \Delta\hat{I}_\alpha(\omega)\Delta\hat{I}_\beta(\omega') + \Delta\hat{I}_\beta(\omega')\Delta\hat{I}_\alpha(\omega)\rangle$ with $\Delta\hat{I}_\alpha(\omega) = \hat{I}_\alpha(\omega) - \langle \hat{I}_\alpha(\omega)\rangle$, where $\hat{I}_\alpha(\omega)$ is the Fourier transform of the current operator at the contact α and $\langle \hat{I}_\alpha(\omega)\rangle$ is its average value. The zero frequency limit will be noted $S_{\alpha,\beta} = S_{\alpha,\beta}(\omega = 0)$. Following the scattering approach, we obtain the noise power,

$$S_{\alpha,\beta} = \frac{e^2}{h}\sum_{\gamma\delta}\sum_{mn} dE\, A_{\gamma\delta}^{mn}(\alpha;E) \times A_{\delta\gamma}^{nm}(\beta;E) \times \left[f_\gamma(E)\{1-f_\delta(E)\} + f_\delta(E)\{1-f_\gamma(E)\}\right], \quad (1)$$

with the notation,

$$A_{\gamma\delta}^{nm}(\alpha;E) = \delta_{mn}\delta_{\alpha\delta}\delta_{\alpha\gamma} - \sum_k S_{\alpha\beta,km}^*(E) S_{\alpha\beta,kn}, \quad (2)$$

with $S_{\alpha\beta,kn}(E)$ the elements of the scattering matrix of the entire system, where α and β denote the contact number and k and n the edge channel.

Since the current is conserved through the fictitious contact 4 (i.e., the current I_4 flowing out of the sample from the contact is zero at any time), its chemical potential μ_4 can fluctuate. This allows to define an average energy distribution $\overline{f_4}(E)$ for electrons in the contact, the shape of which depends on how current conservation through the contact is enforced. This is directly linked to the scattering processes occurring in the contact.

Now, we discuss the current and the noise in the presence of the contact 4. We introduce $G_{\alpha\beta} = \sum_{m=0}^{1}(e^2/h)\, A_{\beta\beta}^{mm}(\alpha;\, E)$. We recall that $I_\alpha = (1/e)\int dE \sum_\beta G_{\alpha\beta} f_\beta + \delta I_\alpha$, where δI_α is the intrinsic part of the fluctuations. We also have $I_\alpha = \langle I_\alpha\rangle + \Delta I_\alpha$. Therefore $\Delta I_\alpha = \delta I_\alpha + (1/e)\int dE \sum_\beta G_{\alpha\beta}(f_\beta - \overline{f_\beta}) = \delta I_\alpha + (1/e)G_{\alpha\beta}(\mu_4 - \overline{\mu_4})$. Since $\langle I_4\rangle = \Delta I_4 = 0$, we also get $\delta I_4 = -(1/e)G_{44}(\mu_4 - \overline{\mu_4})$, hence

$$\Delta I_\alpha = \delta I_\alpha - \frac{G_{\alpha 4}}{G_{44}} \delta I_4. \tag{3}$$

We finally obtain the redistributed noise measured at contact 3, $S_{33}^{\mathrm{in/qe}} = \langle \Delta I_3^2 \rangle$:

$$S_{33}^{\mathrm{in/qe}} = S_{33} - 2\frac{G_{34}}{G_{44}} S_{34} + \frac{G_{34}^2}{G_{44}^2} S_{44}. \tag{4}$$

For a PNJ at $(\nu_{\mathrm{ug}}, \nu_{\mathrm{g}}) = (2, -2)$, this gives

$$S_{33}^{\mathrm{in/qe}} = 2\frac{e^2}{h} \int dE\, \bar{f}_4(E)\left(1 - \bar{f}_4(e)\right). \tag{5}$$

In the inelastic case, $\bar{f}_4(E)$ is the Fermi distribution with $\overline{\mu_4} = \mu_0 + (eV_{\mathrm{sd}})/2$ (Supplementary Figure 1**c**). Then we obtain $I_3 = e^2 V_{\mathrm{sd}}/h$ and $S_{33}^{\mathrm{in}} = 2e^2 k_{\mathrm{B}} T_{\mathrm{eff}}/h$. In the absence of energy losses towards additional degrees of freedom, the effective temperature T_{eff} of the Fermi distribution is given by the balance between the input power from the contacts and the heat flow carried by the electronic channels leaving the junction (2): $k_{\mathrm{B}} T_{\mathrm{eff}} = |eV_{\mathrm{sd}}|\sqrt{4/3}/\pi$. The Fano factor thus becomes $F^{\mathrm{in}} = \sqrt{4/3}/\pi$. In presence of additional energy losses (for instance due to electron-phonon coupling, see below), T_{eff} and thus the noise gradually decrease to zero with increasing interaction length.

In the quasi-elastic case, not only the total current is zero on average ($I_4 = \int dE\, j_4(E) = 0$) but also the contribution to the current of the states of energy E is zero ($\langle j_4(E) \rangle = 0$). This leads to (Supplementary Figure 1**d**)

$$\bar{f}_4(E) = \frac{\bar{f}_1(E)}{2} + \frac{\bar{f}_2(E)}{2}. \tag{6}$$

From the equations (3) and (4), we obtain the current $I_3 = e^2 V_{\mathrm{sd}}/h$ and the noise $S_{33}^{\mathrm{qe}} = 2(e^2/h)|eV_{\mathrm{sd}}| \times 1/4$. The Fano factor becomes $F^{\mathrm{qe}} = 1/4$.

Length dependence

For the length dependence of the quasi-elastic and inelastic scattering models, we first recall some experimental results obtained at $\nu = 2$ in a two-dimensional electron gas formed in a GaAs/AlGaAs heterojunction (5). In this study, one of the two edge channels is driven out of equilibrium (the resulting energy distribution is a double-step function). After several propagation lengths, the electronic energy distribution is measured. In particular, they introduce an inelastic length L_{in}, which is the length scale for energy exchanges in non-equilibrium distribution. They measured an excess temperature T_{exe}

and showed an exponential length dependence $T_{\mathrm{exe}}(L) \sim e^{-L/L_{\mathrm{in}}}$. We assume a similar behavior of the energy relaxation in our case and expect,

$$S_{33}^{\mathrm{qe}} = \frac{2e^2}{h} |eV_{\mathrm{sd}}| \times \frac{1}{4} e^{-L/L_{\mathrm{in}}}. \tag{7}$$

A similar decrease of the noise with the PNJ length can be obtained by the models described in Supplementary Figure 2, where the PNJ is described as a series of fictitious contacts, each of them electrically connected to a (hot or cold, depending on the model) voltage probe. Supplementary Figure 2**a** describes a quasi-elastic scattering case with energy losses towards cold external states, where the energy distribution function of the n-th fictitious contact along the PNJ is determined by an energy-conserving current balance [similar to equation (6)] between the fictitious contact, its next neighbors, and an inelastic voltage probe at zero temperature. The coupling to the cold voltage probe is parametrized by the transmission τ. This yields a gradual transformation of the double-step energy distribution function generated in the first fictitious contact to a zero temperature Fermi function.

Supplementary Figure 2**b** describes a fully inelastic scattering with energy losses towards cold external states: here the fictitious contacts are replaced by inelastic voltage probes described by Fermi functions with a finite temperature given by the power balance between a given voltage probe, its next neighbors, and a zero-temperature voltage probe. The temperature of the first voltage probe is given by the Joule heating dissipated in the junction. Note that the premise of this model is somewhat unrealistic, as it completely disregards the mechanism driving the expected double-step energy distribution function towards a hot Fermi function.

Finally, Supplementary Figure 2**c** describes a model including both energy transfers between modes propagating in the PNJ and energy losses towards cold external states. This is done by coupling, via an electronic transmission coefficient τ_{h}, each of the fictitious contacts to a finite temperature voltage probe which is itself coupled (via a transmission coefficient τ_{c}) to a zero-temperature voltage probe. The energy distribution function in the n-th finite temperature voltage probe is a Fermi function with a temperature T_n determined by the heat balance between the hot probe, the cold probe, and the n-th fictitious contact. The energy distribution of this latter contact is then determined by a current balance, as in the case described in Supplementary Figure 2**a**.

In all the models, the noise is computed from the energy distribution function of the last fictitious contact $S = \int dE\, f_N(E)(1 - f_N(E))$. The evolution of the Fano factor with

the number of fictitious contacts (and thus the length in arbitrary units) is shown in Supplementary Figure 2**d**. Given the proper transmission parameters, all the models yield a length dependence, which is close (but not strictly equal) to the exponential decay with which the experimental data have been fitted in the main text. In particular, the difference between the results of the first and the third model (which would underline the influence of energy relaxation between modes propagating in the junction) is much smaller than the experimental uncertainties.

Relaxation through electron-phonon coupling

We consider energy relaxation mediated by electron-phonon coupling in the PNJ. To model it, we calculate the effective temperature T_{eff} used in the energy distribution function of the fictitious contact 4 in the inelastic case using the standard power balance,

$$J_{\mathrm{IN}} = J_{\mathrm{OUT}}^{\mathrm{el}} + J_{\mathrm{OUT}}^{\mathrm{e-ph}}, \tag{8}$$

with $J_{\mathrm{IN}} = V_{\mathrm{sd}}^2|\nu_1||\nu_2|/2(|\nu_1| + |\nu_2|) = V_{\mathrm{sd}}^2/2$ the Joule power dissipated in the PNJ, $J_{\mathrm{OUT}}^{\mathrm{el}} = \pi^2/6k_{\mathrm{B}}^2T^2(|\nu_1| + |\nu_2|) = 4\pi^2/6k_{\mathrm{B}}^2T^2$ the heat flow carried by the edge channels leaving the PNJ (2), and $J_{\mathrm{OUT}}^{\mathrm{e-ph}} = \Sigma_{\mathrm{e-ph}}LwT_{\mathrm{eff}}^3$ the heat flow transferred to the phonon bath (assumed at zero temperature). L and w~200 nm are respectively the length and the width of the PNJ, and $\Sigma_{\mathrm{e-ph}}$ is the electron-phonon coupling constant on the order of ~1 pW µm^{-2} K^{-3} (5). Note that this very simple model describes the PNJ as a 2D homogeneous area.

Supplementary Figure 3 shows typical results of this calculation. The results qualitatively agree with our observation: the slope of the noise as a function of V_{sd} clearly decreases with increasing L. However, a quantitative analysis of the slope dependence for several values of $\Sigma_{\mathrm{e-ph}}$ shows that this model fails to describe our results (Supplementary Figure 3**b**). Moreover, the presence of a non-negligible heat transfer to phonons is generally indicated by a sublinear behavior of the noise for large enough V_{sd}, which we do not observe at our experimental accuracy.

One can explain this discrepancy with the following arguments: first, treating the PNJ as an homogeneous is obviously incorrect, since by definition the electronic density drastically changes in such a region. Second, a 2D model (even assuming an average density or electron-phonon coupling constant) ignores the particular physics of QH edge channels, especially the suppression of backscattering. As the electron-phonon coupling is generally assumed to increase with impurity scattering, one expects it to be strongly

suppressed in a ballistic edge channel. As such, our results tend to suggest that even though they interact strongly in the PNJ, the "quantum Hall" nature of the copropagating edge channels is preserved in the PNJ. Nonetheless, the presence of relaxation mediated by electron-phonon coupling cannot be completely ruled out in our system, and, provided a more realistic model, perhaps explain the fluctuations in the noise shown in Fig. 3 of the main paper.

Coherent scattering in copropagating edge channels

In this part, we discuss the effects of coherent scattering between copropagating edge states on transport and noise in a system represented in Supplementary Figure 4. We discard the fictitious contact 4 mentioned above, so that, in the PNJ (central region of Supplementary Figure 4**b**), charges flowing from one of the four copropagating edge channels are coherently scattered into the other edge channels. Importantly, the current and noise only depend on the number of copropagating edge channels in the PNJ, the contact from which each of those edge channel stem, and the mechanism coupling those channels. Inter-channel coherent scattering is described by the scattering matrix $S(\epsilon)$, where a state entering the PNJ on a given channel tunnels into the other channels with the same amplitude $\sqrt{\epsilon}$ but with different phases:

$$S = \begin{pmatrix} i\sqrt{1-3\epsilon} & -\sqrt{\epsilon} & -\sqrt{\epsilon} & -\sqrt{\epsilon} \\ -\sqrt{\epsilon} & i\sqrt{1-3\epsilon} & -i\sqrt{\epsilon} & i\sqrt{\epsilon} \\ -\sqrt{\epsilon} & i\sqrt{\epsilon} & i\sqrt{1-3\epsilon} & -i\sqrt{\epsilon} \\ -\sqrt{\epsilon} & -i\sqrt{\epsilon} & i\sqrt{\epsilon} & i\sqrt{1-3\epsilon} \end{pmatrix}. \tag{9}$$

Note that to ensure normalization, ϵ cannot be larger than 1/3. This matrix is then encompassed into the elements $s_{\alpha\beta,\mathrm{kn}}(E)$ of the scattering matrix of the entire system. For example, $s_{31,33}(E)$, which is the amplitude that an electron injected in the edge channel 3 from the contact 1 arrives at the contact 3 in the same edge channel, is given by $i\sqrt{1-3\epsilon}$. At zero temperature, we get the noise autocorrelation $S_{33}^{\mathrm{coh}} = (4e^2/h)|eV_{\mathrm{sd}}|2\epsilon(1-2\epsilon)$. The calculation of the current reaching the contact 3 yields $I_3 = 2V_{\mathrm{sd}}(1-2\epsilon)e^2/h$. For a perfect coherent mode mixing $\epsilon = 1/4$, one has $S_{33}^{\mathrm{coh}} = (e^2/h)|eV_{\mathrm{sd}}|$ and $I_3 = V_{\mathrm{sd}}e^2/h$. Then Fano factor becomes $F^{\mathrm{coh}} = 1/2$.

We now discuss the effect of the PNJ length on the coherent scattering. Increasing L will induce decoherence that we must take into account in the coherent scattering approach. To model decoherence, we introduce a fluctuating phase $\varphi_k(t)$ each scattering process being now described by an amplitude $\sqrt{\epsilon}e^{i\varphi_k(t)}$. Decoherence can be described by a succession of coherent scatterer in series. After N scatterers, the element

of the scattering amplitude becomes $s(t) = \sqrt{\epsilon} e^{\Sigma_k i\varphi_k(t)}$. If the source of decoherence is a Gaussian stochastic process of zero mean, $\varphi_k(t)$ is a Gaussian random variable with $\langle \varphi_k(t) \rangle_\varphi = 0$ and $\langle s(t) \rangle_\varphi$ can be expressed in terms of variance of the phase (3),

$$\langle s(t) \rangle_\varphi = e^{-N\langle\varphi\rangle^2/2}. \tag{10}$$

Therefore, the expected noise becomes,

$$S_{33}^{\text{coh}} = \frac{4e^2}{h}|eV_{\text{sd}}|2\epsilon(1-2\epsilon)e^{-N\langle\varphi\rangle^2/2} = \frac{4e^2}{h}|eV_{\text{sd}}|2\epsilon(1-2\epsilon)e^{-L/L_\varphi}, \tag{11}$$

where L_φ is the coherence length of the system.

Supplementary Methods

We prepared a graphene wafer by thermal decomposition of a 6H-SiC(0001) substrate (6). As a result of doping from the SiC substrate and the HSQ layer, graphene has *n*-type carriers with the density of about 5×10^{11} cm^{-2}.

In our devices, the top gate does not overlap with ohmic contacts to avoid a gate leakage. As a result, ungated regions present between the gated region and ohmic contacts on the gated side. To obtain a good contact to hole edge channels in the presence of the ungated regions, we etched graphene in a comb shape around C_{in}^{h}, where four PNJs are formed in series (Supplementary Figure 5). When the chemical potential of incoming hole edge channels and C_{in}^{h} are μ_0 and $\mu_0 + eV_{\text{sd}}$, respectively, the mode mixing and partitioning in a PNJ lead to the chemical potential $\mu_0 + eV_{\text{sd}}/2$. Undergoing the same process four times, the chemical potential of the outgoing hole edge channel becomes,

$$\mu_{\text{OUT}} = \mu_0 + \left\{1 - \left(\frac{1}{2}\right)^4\right\} eV_{\text{sd}}. \tag{10}$$

Small difference from $\mu_0 + eV_{\text{sd}}$ causes an error of several percent for the estimation of the bias between the electron and hole edge channels. This error does not affect our discussions.

Supplementary References

1. C. Texier, M. Büttiker, Effect of incoherent scattering on shot noise correlations in the quantum Hall regime. Phys. Rev. B 62, 7454-7458 (2000).
2. D. A. Abanin, L. S. Levitov, Quantized Transport in Graphene p-n Junctions in Magnetic Field. Science 317, 641-643 (2007).
3. F. Marquardt, C. Bruder, Influence of Dephasing on Shot Noise in an Electronic Mach-Zehnder Interferometer. Phys. Rev. Lett. 92, 056805 (2004).
4. K. C. Fong et al., Measurement of the electronic thermal conductance channels and heat capacity of graphene at low temperature. Phys. Rev. X 3, 041008 (2013).
5. H. le Sueur, C. Altimiras, U. Gennser, A. Cavanna, D. Mailly, F. Pierre, F. Energy Relaxation in the Integer Quantum Hall Regime. Phys. Rev. Lett. 105, 056803 (2010).
6. S. Tanabe, Y. Sekine, H. Kageshima, M. Nagase, H. Hibino, Half-integer quantum Hall effect in gate-controlled epitaxial graphene devices. Appl. Phys. Express 3, 075102 (2010).
